# Effects of imeglimin on mitochondrial function, AMPK activity, and gene expression in hepatocytes

**DOI:** 10.1038/s41598-023-27689-y

**Published:** 2023-01-13

**Authors:** Kaori Hozumi, Kenji Sugawara, Takaya Ishihara, Naotada Ishihara, Wataru Ogawa

**Affiliations:** 1 Division of Diabetes and Endocrinology, Department of Internal Medicine, Kobe University Graduate School of Medicine, 7-5-1 Kusunoki-cho, Chuo-ku, Kobe, 650-0017 Japan; 2 Department of Biological Sciences, Graduate School of Science, Osaka University, Toyonaka, Japan

**Keywords:** Diabetes, Endocrine system and metabolic diseases

## Abstract

Imeglimin is a recently launched antidiabetic drug structurally related to metformin. To provide insight into the pharmacological properties of imeglimin, we investigated its effects on hepatocytes and compared them with those of metformin. The effects of imeglimin on mitochondrial function in HepG2 cells or mouse primary hepatocytes were examined with an extracellular flux analyzer and on gene expression in HepG2 cells by comprehensive RNA-sequencing analysis. The effects of the drug on AMPK activity in HepG2 cells, mouse primary hepatocytes, and mouse liver were also examined. Treatment of HepG2 cells or mouse primary hepatocytes with imeglimin reduced the oxygen consumption rate coupled to ATP production. Imeglimin activated AMPK in these cells whereas the potency was smaller than metformin. Bolus administration of imeglimin in mice also activated AMPK in the liver. Whereas the effects of imeglimin and metformin on gene expression in HepG2 cells were similar overall, the expression of genes encoding proteins of mitochondrial respiratory complex III and complex I was upregulated by imeglimin but not by metformin. Our results suggest that imeglimin and metformin exert similar pharmacological effects on mitochondrial respiration, AMPK activity, and gene expression in cultured hepatocytes, whereas the two drugs differ in their effects on the expression of certain genes related to mitochondrial function.

## Introduction

Imeglimin is a recently launched antidiabetic drug that is structurally related to metformin^1,2^. Despite the structural similarity of the two drugs, however, imeglimin appears to exert its antidiabetic action by a mechanism that differs from that of metformin. Imeglimin augments glucose-induced insulin secretion by pancreatic beta cells^3–8^. Administration of imeglimin thus increases insulin secretion during an oral glucose tolerance test and a hyperglycemic clamp in rats^5,6^ and humans^3^, respectively. Although the mechanism underlying this effect of imeglimin remains unclear, an NAD^+^–cyclic ADP-ribose–Ca^2+^ signaling pathway has been implicated^8^. The antidiabetic action of imeglimin is thus thought to be mediated primarily at the level of the pancreas.

The antidiabetic action of metformin, on the other hand, is largely attributable to the suppression of gluconeogenesis in the liver^9^. Metformin increases the AMP/ATP ratio in hepatocytes by inhibiting mitochondrial respiratory chain complex I, which results in the activation of AMP-activated protein kinase (AMPK)^9^. Evidence suggests that AMPK mediates the antidiabetic action of metformin^10–13^, although some findings have challenged this notion^14–16^. The inhibition of mitochondrial respiration does appear to contribute to the antidiabetic action of metformin in other ways, however^17,18^.

Given its structural similarity to metformin, the effects of imeglimin on the liver have also been investigated. Administration of imeglimin to mice with diet-induced obesity ameliorated hepatic steatosis^6^, with a similar effect having been observed with metformin^16,19^. Moreover, imeglimin was found to reduce both the ATP/ADP ratio and glucose production in cultured hepatocytes, as does metformin^20^. Unlike metformin, however, imeglimin did not inhibit the activity of respiratory chain complex I in hepatocytes^20^. Instead, treatment of obese mice with imeglimin increased the mRNA abundance and the activity of enzymes of respiratory chain complex III in the liver^6^. These findings thus indicated that the two drugs have some similar but also some different effects in hepatocytes.

To provide further insight into the pharmacological properties of imeglimin, we have now investigated its effects in hepatocytes and compared them with those of metformin. We here show that imeglimin has similar effects on mitochondrial respiration, AMPK activity, and gene expression as does metformin, but that some effects of imeglimin differ from those of metformin.

## Results

### Effects of imeglimin and metformin on mitochondrial respiration and ROS production in HepG2 cells and mouse primary hepatocytes

**Figure 1:**
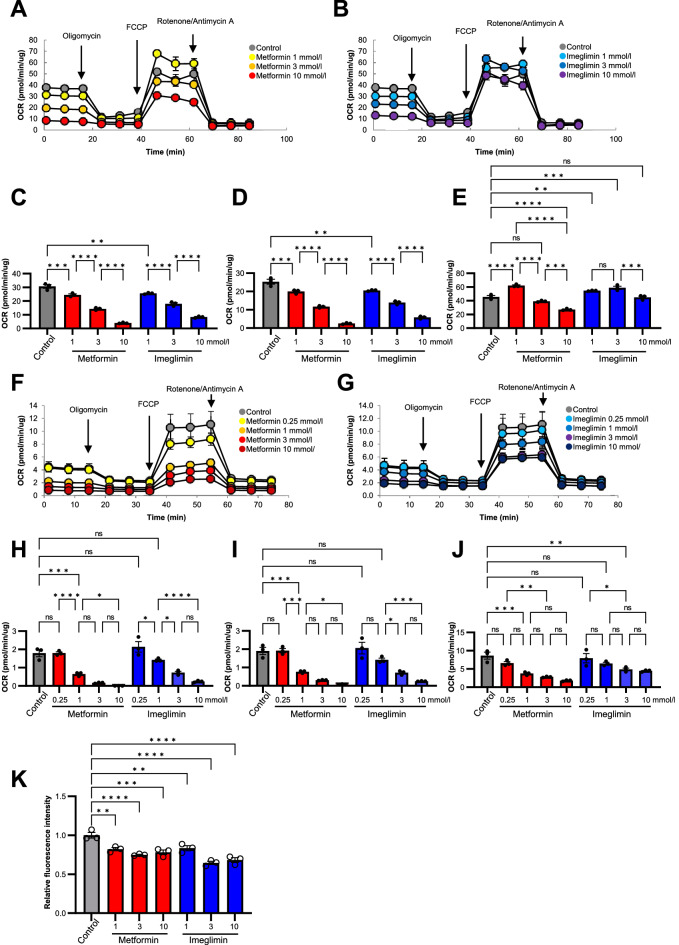
Effects of imeglimin and metformin on mitochondrial respiration and ROS production in HepG2 cells and mouse primary hepatocytes. (**A**, **B**) OCR of HepG2 cells after stimulation with vehicle (control) or with metformin (**A**) or imeglimin (**B**) at 1, 3, or 10 mmol/l for 3 h. (**C**–**E**) Basal respiration (the difference between the last rate measurement before oligomycin addition and the minimum rate measurement after rotenone/antimycin A addition) (**C**), respiration coupled to ATP production (the difference between the last rate measurement before oligomycin addition and the minimum rate measurement after oligomycin addition) (**D**), and maximal respiration (the difference between the maximum rate measurement after FCCP addition and the minimum rate measurement after oligomycin addition) (**E**) for the cells in (**A**) and (**B**). (**F**, **G**) OCR of mouse primary hepatocytes after stimulation with vehicle (control) or with metformin (**F**) or imeglimin (**G**) at 0.25, 1, 3, or 10 mmol/l for 3 h. (**H**–**J**) Basal respiration (**H**), respiration coupled to ATP production (**I**), and maximal respiration (**J**) for the cells in (**F**) and (**G**). (**K**) ROS of HepG2 cells after stimulation with vehicle (control) or with metformin (**A**) or imeglimin (**B**) at 1, 3, or 10 mmol/l for 12 h. All quantitative data are means ± SEM (*n* = 3 independent experiments). *p < 0.05, **p < 0.01, ***p < 0.001, ****p < 0.0001, ns (one-way ANOVA followed by Tukey’s test) for (**C**–**E, H**–**K**).

We first examined the effects of imeglimin and metformin on mitochondrial respiratory function with the use of an extracellular flux analyzer. Treatment of the human hepatoma cell line HepG2 with metformin at concentrations of 1, 3, or 10 mmol/l for 3 h resulted in a concentration-dependent decrease in the oxygen consumption rate (OCR) under the basal condition (Fig. 1A, C) as well as in that coupled with ATP production (Fig. 1A, D). Treatment of the cells with imeglimin at the same concentrations as metformin also reduced both these parameters of mitochondrial function in a concentration-dependent manner (Fig. 1B–D). Maximal mitochondrial respiration was increased by exposure of the cells to metformin at 1 mmol/l and decreased by that to metformin at 10 mmol/l (Fig. 1A, E). Treatment of the cells with 1 or 3 mmol/l imeglimin also increased this parameter, whereas treatment with the drug at 10 mmol/l had no significant effect compared with vehicle.

We also examined the effects of the drugs on mitochondrial respiratory function in mouse primary hepatocytes. Metformin and imeglimin inhibited the OCR under the basal condition and that coupled with ATP production as well as maximal mitochondrial respiration in a concentration-dependent manner (Fig. 1F–J). The inhibitory effects of metformin on mitochondrial respiratory function appeared greater than those of imeglimin particularly in mouse primary hepatocytes (Fig. 1C–E, H–J).

Given the mitochondrial electrons chain is a source of reactive oxygen species (ROS)^21,22^, we examined the effects of these drugs on the intracellular ROS contents. Treatment of HepG2 cells with metformin or imeglimin reduced ROS contents at concentrations as low as 1 mmol/l, and the increase in the drug concentration did not further reduce the ROS contents (Fig. 1K).

These results indicated that imeglimin and metformin exert similar inhibitory effects on mitochondrial respiratory function and the intracellular ROS contents whereas the effects of metformin on the former parameter is greater than those of imeglimin. These drugs may also possess stimulatory effects on maximal mitochondrial respiration at certain concentrations in HepG2 cells.

### Effects of imeglimin and metformin on AMPK activity in HepG2 cells and mouse primary hepatocytes

**Figure 2:**
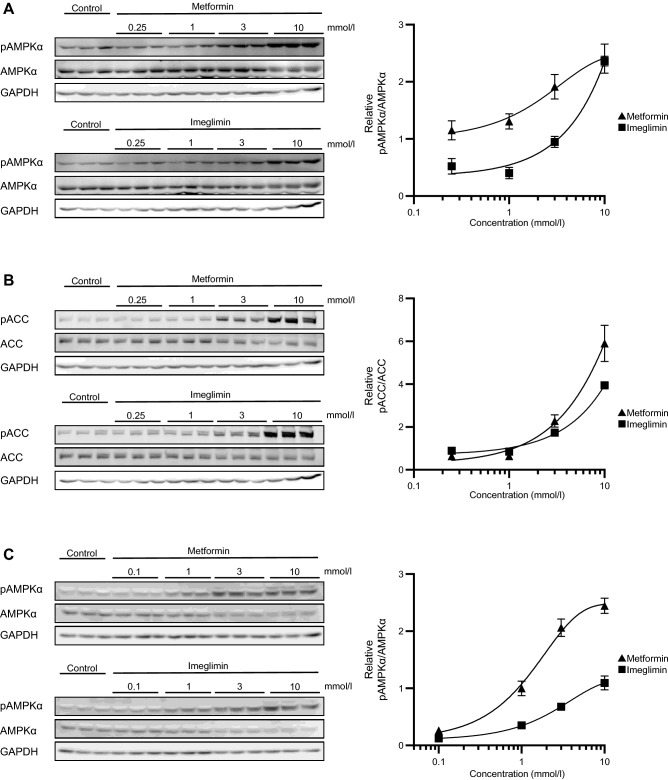
Effects of imeglimin and metformin on AMPK activity in HepG2 cells and mouse primary hepatocytes (**A**, **B**) Immunoblot analysis of phosphorylated (p) and total forms of AMPKα (**A**) and of ACC (**B**) in HepG2 cells treated with vehicle (control) or with 0.25, 1, 3, or 10 mmol/l metformin or imeglimin for 3 h. Blots are shown on the left, and quantitative data for the pAMPKα/AMPKα or pACC/ACC ratio from three independent experiments on the right. (**C**) Immunoblot analysis of phosphorylated (p) and total forms of AMPKα in mouse primary hepatocytes treated with vehicle (control) or with 0.1, 1, 3, or 10 mmol/l metformin or imeglimin for 3 h. Blots are shown on the left, and quantitative data for the pAMPKα/AMPKα ratio from three independent experiments on the right. Original blots are presented in Fig. S2.

Given that metformin-induced inhibition of ATP production is related to the activation of AMPK, we next investigated the effect of imeglimin on this serine-threonine kinase. Treatment of HepG2 cells or mouse primary hepatocytes with metformin or imeglimin for 3 h induced phosphorylation of AMPKα at Thr^172^, which reflects the activity of this kinase, in a concentration-dependent manner (Figs. 2A, C, S2A–D, I–L). The phosphorylation of ACC, an endogenous substrate of AMPK, was also stimulated by both drugs in HepG2 cells (Figs. 2B, S2E–H), confirming that AMPK is activated in these cells. We quantitated the intensity of the bands with antibodies to the phosphorylated proteins with those with antibodies to non-phosphorylated AMPK or ACC (Fig. 2A–C) or to GAPDH (Fig. S1A–C) as internal standards. The quantitative analyses revealed that the effect of metformin on the phosphorylation of AMPKα was greater than those of imeglimin in primary hepatocytes (Figs. 2C, S1C).

**Figure 3:**
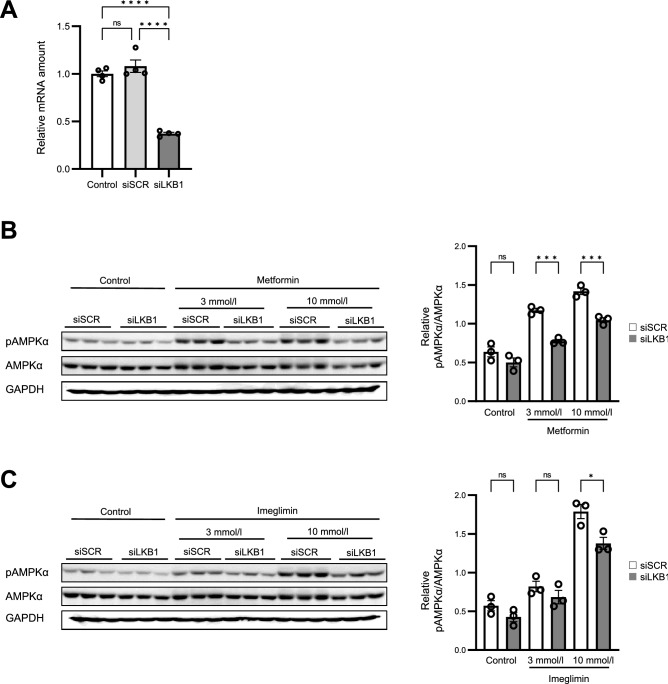
LKB1-mediated phosphorylation of AMPK induced by imeglimin and metformin in HepG2 cells. (**A**) RT-qPCR analysis of LKB1 mRNA in cells transfected or not (control) with scrambled (siSCR) or LKB1 (siLKB1) siRNAs for 72 h. Data are from four independent experiments. (**B**, **C**) Immunoblot analysis of phosphorylated and total forms of AMPKα in cells transfected with LKB1-targeting or control siRNAs for 72 h and then stimulated with vehicle or with 3 or 10 mmol/l metformin or imeglimin for 3 h. Blots are shown on the left, and quantitative data for the pAMPKα/AMPKα ratio from three independent experiments on the right. Original blots are presented in Fig. S3. All quantitative data are means ± SEM. *p < 0.05, **p < 0.01, ***p < 0.001, ****p < 0.0001, ns as determined either by one-way ANOVA followed by Tukey’s test (**A**) or by Student’s *t* test (**B**, **C**).

The serine-threonine kinase LKB1 (liver kinase B1) directly phosphorylates and activates AMPKα in response to metformin treatment^10,11^. Transfection of HepG2 cells with siRNAs specific for LKB1 resulted in an ~ 67% reduction in the amount of *LKB1* mRNA (Fig. 3A). Such transfection inhibited the phosphorylation of AMPKα induced by metformin or imeglimin (Figs. 3B, C, S3). Quantitative analyses with the use of AMPKα (Fig. 3B, C) or GAPDH (Fig. S1D, E) as internal standards revealed that the siRNAs significantly attenuated the stimulatory effects of 3 or 10 mmol/l metformin and 10 mmol/l imeglimin on AMPKα phosphorylation (Figs. 3B, C, S1D, E). These results thus suggested that imeglimin activates AMPK at least in part in a manner dependent on LKB1.

### Effects of imeglimin and metformin on AMPK activity in mouse liver

**Figure 4:**
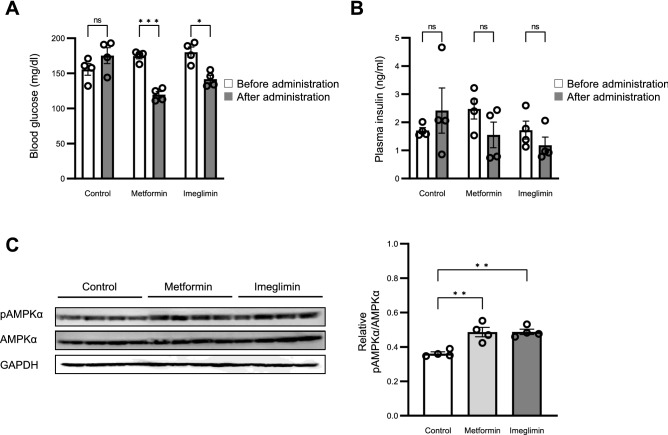
Phosphorylation of AMPKα induced by imeglimin and metformin in the liver in vivo. (**A**, **B**) Blood glucose (**A**) and plasma insulin (**B**) concentrations at 1 h after i.p. injection of vehicle (control) or of metformin or imeglimin at 250 mg/kg in mice that had been maintained on a standard diet, deprived of food overnight, and allowed to refeed for 5 h. (**C**) Immunoblot analysis of phosphorylated and total forms of AMPKα in the liver of mice treated as in (**A**) and (**B**). Original blots are presented in Fig. S4. All quantitative data are means ± SEM (*n* = 4 mice). *p < 0.05, **p < 0.01, ***p < 0.001, ****p < 0.0001, ns by Student’s *t* test (**A**, **B**) or one-way ANOVA followed by Tukey’s test (**C**).

We next investigated whether imeglimin activates AMPK in the liver of mice. Imeglimin or metformin was administered i.p. to C57BL/6J mice at a dose of 250 mg/kg. Blood glucose levels were significantly reduced at 1 h after administration of each drug (Fig. 4A), whereas plasma insulin levels were unaffected by injection of either agent (Fig. 4B). Phosphorylation of AMPKα in the liver of mice treated with metformin or imeglimin was significantly increased compared with that in the liver of vehicle-treated animals (Figs. 4C, S4). Quantitative analyses with non-phosphorylated AMPKα (Fig. 4C) or GAPDH (Fig. S1F) as internal standards revealed that there is no significant difference in the effects of the two drugs on the phosphorylation of AMPK. These results thus indicated that imeglimin activates AMPK in the liver of living animals.

### Effects of imeglimin and metformin on gene expression in HepG2 cells

**Figure 5:**
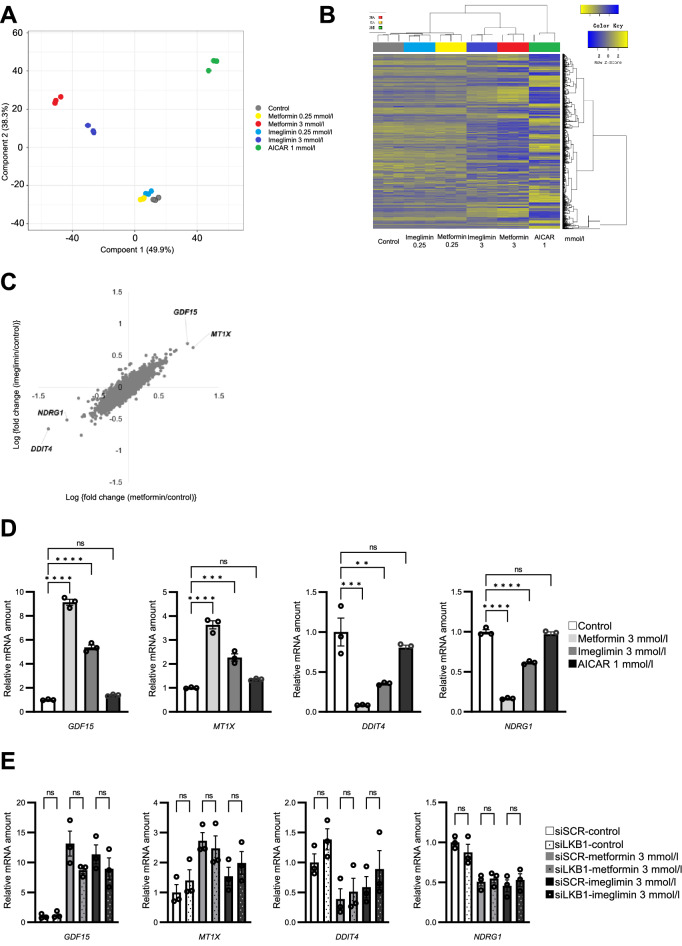
Similarities and differences in the effects of imeglimin and metformin on gene expression in HepG2 cells. (**A**–**C**) HepG2 cells were stimulated with 0.25 or 3 mmol/l metformin or imeglimin, with 1 mmol/l AICAR, or with vehicle for 12 h, after which RNA was isolated from the cells and subjected to RNA-seq analysis (*n* = 3 independent replicates). Multidimensional scaling analysis (**A**) and hierarchical clustering analysis (**B**) of the sequencing data, as well as a logarithmic scatter plot of the fold changes in gene expression (TPM > 10) induced by metformin or imeglimin at 3 mmol/l relative to the vehicle control (**C**), are shown. (**D**) RT-qPCR analysis of expression of the four genes indicated in (**C**) for cells treated with 3 mmol/l metformin or imeglimin, with 1 mmol/l AICAR, or with vehicle. (**E**) RT-qPCR analysis of expression of the four genes indicated in (**C**) for cells transfected with LKB1-targeting or control siRNAs for 72 h and then stimulated with vehicle or with 3 mmol/l metformin or imeglimin for 12 h. All RT-qPCR data are means ± SEM (*n* = 3 independent replicates). *p < 0.05, **p < 0.01, ***p < 0.001, ****p < 0.0001, ns (one-way ANOVA followed by Tukey’s test).

We finally investigated the effects of imeglimin and metformin on gene expression in HepG2 cells. The cells were treated with the drugs for 12 h and were then subjected to comprehensive RNA-seq analysis. Multidimensional scaling revealed that treatment of the cells with either drug at a concentration of 0.25 mmol/l had only small effects on gene expression, whereas both drugs induced substantial changes in a similar direction at a concentration of 3 mmol/l (Fig. 5A). On the other hand, treatment of the cells with the AMPK activator AICAR induced changes in gene expression in a different direction (Fig. 5A). Hierarchical clustering analysis revealed similar results, with metformin and imeglimin having only minor effects on the heat map pattern at 0.25 mmol/l and more substantial effects at 3 mmol/l that were similar for both drugs but different from those of AICAR (Fig. 5B). These results suggested that the overall effects of imeglimin and metformin on gene expression were similar and that these effects were largely independent of their abilities to activate AMPK.

A scatter diagram comparing the changes in gene expression induced by metformin and by imeglimin again showed that the effects of the two drugs at 3 mmol/l were generally similar (Fig. 5C). Genes prominently upregulated by both drugs included *GDF15* and *MT1X*, and those prominently downregulated included *DDIT4* and *NDRG1*. The changes in the expression of these four genes by metformin and imeglimin were confirmed by RT-qPCR analysis (Fig. 5D); AICAR however did not affect the expression of these genes (Fig. 5D). We further investigated the effects of siRNAs for LKB1 on the changes in the expression these four genes induced by metformin or imeglimin. The introduction of the siRNA did not attenuate the effects of the drugs on the expression of these four genes (Fig. 5E). These results collectively suggest that the effects of metformin and imeglimin on the expression of these four genes were independent of AMPK.

**Figure 6:**
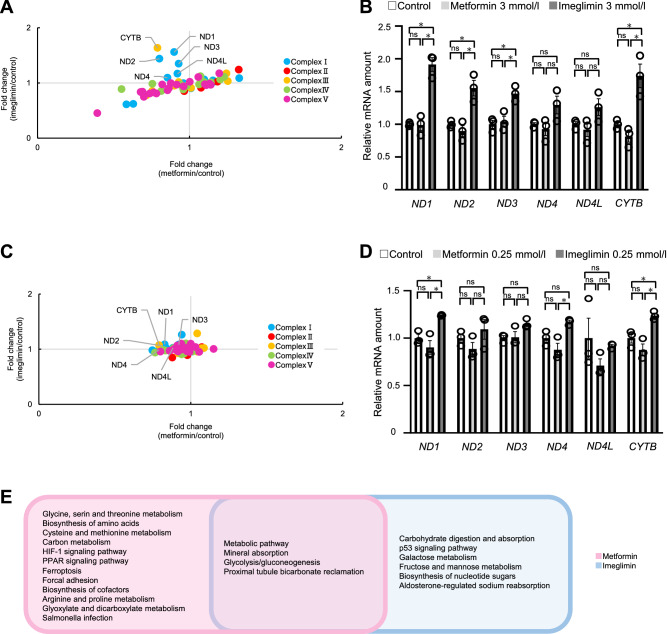
Differences in the effects of imeglimin and metformin on genes encoding proteins of mitochondrial respiratory complexes in HepG2 cells. (**A**) Scatter plot of RNA-seq data in Fig. 5 for the fold change in expression of genes encoding proteins of mitochondrial respiratory complexes induced by metformin or imeglimin at 3 mmol/l relative to the vehicle control. (**B**) RT-qPCR analysis of expression of the six genes indicated in (**A**) for cells treated with metformin or imeglimin at 3 mmol/l. (**C**) Scatter plot of RNA-seq data in Fig. 5 for the fold change in expression of genes encoding proteins of mitochondrial respiratory complexes induced by metformin or imeglimin at 0.25 mmol/l relative to the vehicle control. (**D**) RT-qPCR analysis of expression of the six genes indicated in (**C**) for cells treated with metformin or imeglimin at 0.25 mmol/l. (**E**) KEGG pathway analysis for genes whose expression was affected by metformin or imeglimin alone or by both drugs at 3 mmol/l. All RT-qPCR data are means ± SEM (*n* = 3 independent replicates). *p < 0.05, **p < 0.01, ***p < 0.001, ****p < 0.0001, ns (one-way ANOVA followed by Tukey’s test).

Given that the effects of imeglimin and metformin on the activity of mitochondrial respiratory complexes have previously been shown to differ^20^ and that imeglimin was previously shown to upregulate the expression and activity of certain respiratory complex enzymes in mouse liver^6^, we investigated the effects of the two drugs on the expression of genes encoding mitochondrial respiratory complexes. The list of genes for components of each mitochondrial respiratory complex and the changes in their expression triggered by metformin or imeglimin at 3 mmol/l are shown in Table S1. The expression of most of these genes was up- or downregulated similarly by both drugs (Fig. 6E). However, *CYTB* (encoding cytochrome b of complex III) and *ND1*, *ND2*, and *ND3* (encoding NADH-ubiquinone oxidoreductase chains 1, 2, and 3, respectively, of complex I) were downregulated by metformin but upregulated by imeglimin (Fig. 6A), suggesting that the two drugs influence some aspects of mitochondrial function differently. RT-qPCR analysis confirmed that the expression of these genes was upregulated only by imeglimin, with the inhibitory effects of metformin being minimal (Fig. 6B). We also examined the effects on the expression of these six genes at a lower concentration of the drugs (Fig. 6C). Treatment of the cells with 0.25 mmol/l of imeglimin increased only the expression of *ND1* and *CYTB* whereas that with metformin did not affect the expression of any genes we tested (Fig. 6D).

KEGG pathway analysis revealed that the expression of genes related to “metabolic pathway,” “mineral absorption,” “glycolysis/gluconeogenesis,” and “proximal tubule bicarbonate reclamation” was markedly affected by both metformin and imeglimin at 3 mmol/l (Fig. 6E). Various other pathways were significantly affected by either of the two drugs.

## Discussion

We here investigated the differences and similarities in the biochemical properties of imeglimin and metformin, one of the newest and the oldest antidiabetic drugs, respectively. The effects of these two agents on mitochondrial function and intracellular ROS contents in cultured hepatocytes were similar overall, and they both activated AMPK both in the cultured cells and in the liver of live mice. Moreover, the effects of imeglimin and metformin on gene expression in HepG2 cells were generally similar. These results thus indicated that the two drugs share similarities not only in chemical structure but also in biochemical properties with regard to their effects on hepatocytes.

Metformin inhibits mitochondrial respiratory chain complex I^23,24^, which leads to a decline in the intracellular ATP concentration and to consequent activation of AMPK^10,25^. We have now shown that imeglimin reduced the levels of both basal mitochondrial respiration and mitochondrial respiration linked to ATP production in hepatocytes, as did metformin. These results are consistent with a previous finding that imeglimin reduced the ATP/ADP ratio in primary cultured hepatocytes^20^. The mechanism by which imeglimin inhibits such mitochondrial function is currently unknown. Imeglimin was also previously found not to inhibit the activities of complex I or complex V in hepatocytes, whereas metformin inhibited both^20^. The mitochondrial respiratory complex components targeted by imeglimin in its inhibition of basal respiration and ATP production remain to be identified.

AMPK is activated in response to an increase in the intracellular AMP/ATP ratio^26^ and exerts various effects on energy metabolism. Activation of AMPK is thought to contribute to the glucose-lowering effect of metformin in multiple ways, whereas AMPK-independent mechanisms have also been implicated in the drug action^9^. We have here shown that imeglimin activates AMPK. Evidence suggests that metformin actually exerts pleiotropic effects via AMPK, including antitumorigenic^27,28^, antiatherosclerotic^27,29^, and anti-inflammatory actions^27,30,31^. It will thus be of interest to examine whether imeglimin also has such multiple beneficial effects.

Whereas the effects of these two drugs on mitochondrial respiration and AMPK activation were overall similar, the potency of metformin appeared greater than that of imeglimin. Furthermore, the differences between imeglimin and metformin were more apparent in primary hepatocytes than in HepG2 cells. These results are consistent with a previous study showing that the effects of imeglimin on the ATP/ADP ratio and glucose production in primary hepatocytes were smaller than those of metformin (20). Thus, even if imeglimin possesses hepatic actions in vivo, such action**s** might be smaller than those of metformin.

We found that the expression of *GDF15* (growth differentiation factor 15) was markedly increased by imeglimin and by metformin in cultured hepatocytes. Recent studies have suggested that metformin-induced expression of *GDF15* in hepatocytes or intestinal cells has beneficial effects on metabolism that are associated with a reduction in food intake and body weight in mice^32,33^. The expression of *MT1X*, which encodes metallothionein 1, was also prominently induced by both drugs. Metallothionein 1 intercepts heavy metals and thereby protects cells from damage induced by reactive oxygen species. The expression of *MT1X* in hepatoma cells was found to correlate with the extent of malignancy^34,35^. The expression of *NDRG1* (N-Myc downstream regulated gene 4) and *DDIT4* (DNA damage–inducible transcript 4), both of which are also implicated in tumor growth^36,37^, was downregulated by both imeglimin and metformin. Whether these genes are related to the anticancer action of metformin and whether imeglimin possesses similar anticancer effects warrant further investigation.

Despite the general similarity in the effects of imeglimin and metformin on gene expression, the expression of genes encoding components of mitochondrial respiratory chain complex I (NADH-ubiquinone oxidoreductase chains) and III (cytochrome b) was induced only by imeglimin. A previous study found that maintenance of mice on a high-fat and high-sucrose diet inhibited the activity of mitochondrial respiratory chain complex III in the liver and that treatment of the mice with imeglimin reversed this inhibition^6^. Upregulation of the cytochrome b gene might contribute to the beneficial effect of imeglimin on complex III activity. On the other hand, imeglimin treatment suppressed the activity of complex I in the liver of mice fed a high-fat and high-sucrose diet^6^. The biological consequences of the upregulation of NADH-ubiquinone oxidoreductase gene expression by imeglimin remain to be determined.

Whereas the inhibition of mitochondrial function and the activation of AMPK are thought to contribute to the pharmacological actions of metformin, such effects of metformin are apparent in vitro only at much higher concentrations than those achieved in the circulation of living animals treated with pharmacological doses of the drug^24,38^. This discrepancy in drug concentrations argues against a role for the suppression of mitochondrial function in the pharmacological actions of metformin^39,40^. We have now shown that imeglimin inhibits mitochondrial function and activates AMPK at similar concentrations to metformin, which were also much higher than those achieved in the circulation after administration of a pharmacological dose^38,39^. Thus, it is possible that AMPK is not activated in the liver of humans treated with imeglimin or metformin at clinical dose. However, some investigators point out the possibility that cultured cells require higher doses of metformin to respond than living animals via several possible mechanisms^38,39^. It therefore remains unclear whether the effects of imeglimin revealed in our study play a role in its pharmacological actions in the living body.

In summary, we have shown that imeglimin exerts biochemical effects similar to those of metformin on mitochondrial respiration, AMPK activity, and gene expression in hepatocytes at relatively high concentrations. However, effects on the expression of certain genes related to mitochondrial function differed between the two drugs. It will be of interest to study the differences and similarities in the biochemical properties of these drugs with other cell types including muscle cells, pancreatic beta cells, and intestinal cells. Despite intense efforts for more than half a century, the mechanism by which metformin exerts its antidiabetic action remains incompletely understood. Our findings on the differences and similarities of imeglimin and metformin may provide important insight into not only the new drug but also the widely administered and scientifically intriguing old one.

## Methods

### Cell culture

HepG2 cells (ECACC, UK) were maintained under a mixture of 95% air and 5% CO_2_ at 37℃ in six-well plates containing high-glucose (25 mmol/l) DMEM supplemented with 10% heat-inactivated FBS. They were exposed to metformin (Merck, Germany), imeglimin (provided by Sumitomo Pharma Co. Ltd., Japan), or vehicle before analysis.

### siRNA transfection

Cells were transfected for 72 h in six-well plates with 25 nmol/l small interfering RNAs (siRNAs) targeting *LKB1* (*STK11* siRNA-SMART pool, Horizon, UK) or control siRNAs (nontargeting siRNA pool #2, Horizon) and with the use of the Lipofectamine RNAiMAX reagent (Invitrogen, USA). They were then stimulated with metformin or imeglimin for 3 h before immunoblot analysis.

### Primary hepatocytes

Mouse primary hepatocytes were isolated from male C57BL/6J mice (CLEA Japan) by enzymatic digestion with collagenase essentially as described^41–43^. The isolated hepatocytes were seeded at a density of 2.25 × 10^5^ cells per well in 6-well plates (coated with collagen) for immunoblot in William's E Medium with 5% FBS 0.5%, penicillin, and 0.5% streptomycin.

### Mice

This study was performed in accordance with relevant guidelines and regulations. Animal experiments were carried out in accordance with the ARRIVE guidelines and approved by the animal experimentation committee of Kobe University Graduate School of Medicine. C57BL/6J mice were obtained from CLEA Japan and maintained on a normal diet and on a 12-h-light/12-h-dark cycle. At 8 weeks of age, male mice were deprived of food overnight, allowed to refeed for 5 h, and then injected i.p. with vehicle or with metformin or imeglimin at 250 mg/kg. A blood sample was collected from the tail vein at 1 h after the injection for determination of the blood glucose level with a glucose meter (Sanwa Chemical, Japan) and of the plasma insulin concentration with an ELISA kit for mouse insulin (Morinaga, Japan).

### Immunoblot analysis

Cells were solubilized with a lysis buffer consisting of 0.2% Triton X-100, a protease inhibitor cocktail (Merck, Germany), and a phosphatase inhibitor cocktail (Nacalai Tesque, Japan) in PBS. Mouse liver was homogenized in a lysis buffer comprising 20 mmol/l Tris–HCl (pH 7.5), 150 mmol/l NaCl, 2 mmol/l EDTA, 1% Nonidet P-40, 10% glycerol, and protease inhibitor and phosphatase inhibitor cocktails. The protein concentration of each sample was measured with a BCA protein assay kit (Thermo Fisher Scientific, USA), and equal amounts of protein were fractionated by SDS-PAGE. The separated proteins were transferred to a nitrocellulose membrane (Cytiva, Japan), which was then exposed to 3% BSA (Fuji Film Wako, Japan) before incubation with primary antibodies including those to α subunits of AMPK (AMPKα), to phosphorylated AMPKα (Thr^172^), to acetyl-CoA carboxylase (ACC), and to phosphorylated ACC (Ser^79^), all of which were obtained from Cell Signaling Technology (USA), and to GAPDH, from MBL International (USA). Immune complexes were detected with Anti-Rabbit IgG (H + L) HRP Conjugate (Promega, USA) and chemiluminescence reagents (ECL prime, Cytiva) and were quantified with the use of ImageJ software. Original blots are shown in Fig. S2–4.

### Mitochondrial respiration

HepG2 cells in culture medium were seeded at a density of 1.2 × 10^5^ per well in a Seahorse XF^e^24-well plate (Agilent Technologies, USA). After 24 h, the medium was changed to Seahorse XF DMEM containing 10 mmol/l glucose, 1 mmol/l pyruvate, and 2 mmol/l L-glutamine, and the cells were stimulated with metformin or imeglimin for 3 h. Mitochondrial respiratory chain activity was measured with a Seahorse XF^e^24 extracellular flux analyzer. Oxygen consumption was determined under the basal condition and after treatment with oligomycin (2 μmol/l), carbonyl cyanide-*p*-trifluoromethoxyphenylhydrazone (FCCP, 1 μmol/l), and both rotenone (0.5 μmol/l) and antimycin A (0.5 μmol/l), and was normalized by protein concentration measured with a BCA protein assay kit (Thermo Fisher Scientific, USA).

Primary hepatocytes were seeded at a density of 5.0 × 10^3^ per well in a Seahorse XF^e^96-well plate (Agilent Technologies, USA). After 16 h, the medium was changed to Seahorse XF DMEM containing 10 mmol/l glucose, 1 mmol/l pyruvate, and 2 mmol/l L-glutamine, and the hepatocytes were stimulated with metformin or imeglimin for 3 h. Mitochondrial respiratory chain activity was measured with a Seahorse XF^e^96 extracellular flux analyzer. Oxygen consumption was determined under the basal condition and after treatment with oligomycin (2 μmol/l), carbonyl cyanide-*p*-trifluoromethoxyphenylhydrazone (FCCP, 1 μmol/l), and both rotenone (0.5 μmol/l) and antimycin A (0.5 μmol/l), and was normalized by protein concentration measured with a BCA protein assay kit (Thermo Fisher Scientific, USA).

### RNA-seq and RT-qPCR analysis

Cells were incubated in 12-well plates and stimulated with 0.25 or 3 mmol/l metformin or imeglimin, with 1 mmol/l 5-aminoimidazole-4-carboxamide ribonucleotide (AICAR), or with vehicle for 12 h. Total RNA was extracted from the cells with the use of an RNeasy Mini Kit (Qiagen, Germany) and was subjected to RNA-sequencing (RNA-seq) analysis by Macrogen Japan. An mRNA paired-end library was prepared from each RNA sample with the use of a TruSeq Stranded mRNA Library Preparation Kit (Illumina, USA), and each library was sequenced with an Illumina NovaSeq 6000 system. The number of reads was 4 × 10^7^ per sample, and GRCh38 was used as the human reference sequence genome. Kyoto Encyclopedia of Genes and Genomes (KEGG) pathway analysis of genes (https://www.kegg.jp/kegg/kegg1.html) was performed to identify relevant biological pathways with the use of the Database for Annotation, Visualization, and Integrated Discovery (DAVID) v6.8 online tool (https://david.ncifcrf.gov). Genes with expression values of Transcripts Per kilobase Million (TPM) > 10 and with a fold-change >|2| were used for the pathway analysis. P-value less than 0.05 are considered to be statistically significant. For RT and quantitative PCR (qPCR) analysis, isolated RNA was subjected to RT with the use of a high-capacity RT kit (Applied Biosystems, USA) and the resulting cDNA was subjected to qPCR with specific primers (Table S2) in a real-time PCR system (Applied Biosystems, USA). The abundance of target mRNAs was normalized by that of 36B4 mRNA.

### Statistical analysis

Quantitative data are presented as means ± SEM and were analyzed with Student’s *t* test or by one-way ANOVA followed by Tukey’s test. A *p* value of < 0.05 was considered statistically significant.

## Supplementary Information

Supplementary Information.

## Data Availability

Original RNA-seq data have been deposited in the Gene Expression Omnibus database of NCBI (GSE208245). https://www.ncbi.nlm.nih.gov/geo/query/acc.cgi?acc=GSE208245.
