# Supplementary material for: Effects of imeglimin on mitochondrial function, AMPK activity, and gene expression in hepatocytes
Source: Sci Rep. 2023 Jan 13;13:746. doi: 10.1038/s41598-023-27689-y (PMC9839736; doi:10.1038/s41598-023-27689-y)
Supplement: Supplementary file 1 — Supplementary Information. [file 41598_2023_27689_MOESM1_ESM.pptx]

## Slide 1
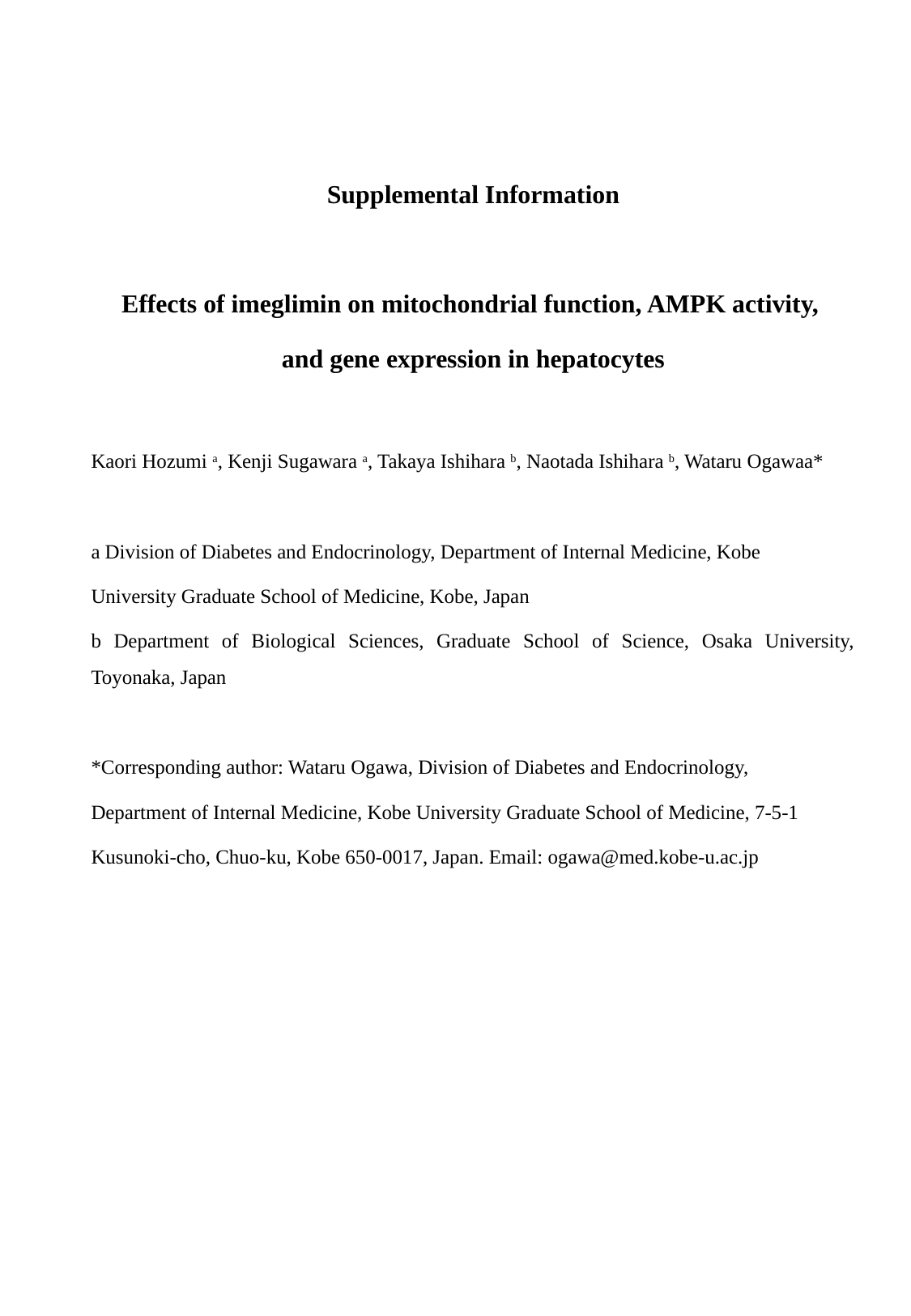

Supplemental Information
Effects of imeglimin on mitochondrial function, AMPK activity,
and gene expression in hepatocytes
Kaori Hozumi a, Kenji Sugawara a, Takaya Ishihara b, Naotada Ishihara b, Wataru Ogawaa*
a Division of Diabetes and Endocrinology, Department of Internal Medicine, Kobe
University Graduate School of Medicine, Kobe, Japan
b Department of Biological Sciences, Graduate School of Science, Osaka University, Toyonaka, Japan
*Corresponding author: Wataru Ogawa, Division of Diabetes and Endocrinology,
Department of Internal Medicine, Kobe University Graduate School of Medicine, 7-5-1
Kusunoki-cho, Chuo-ku, Kobe 650-0017, Japan. Email: ogawa@med.kobe-u.ac.jp

## Slide 2
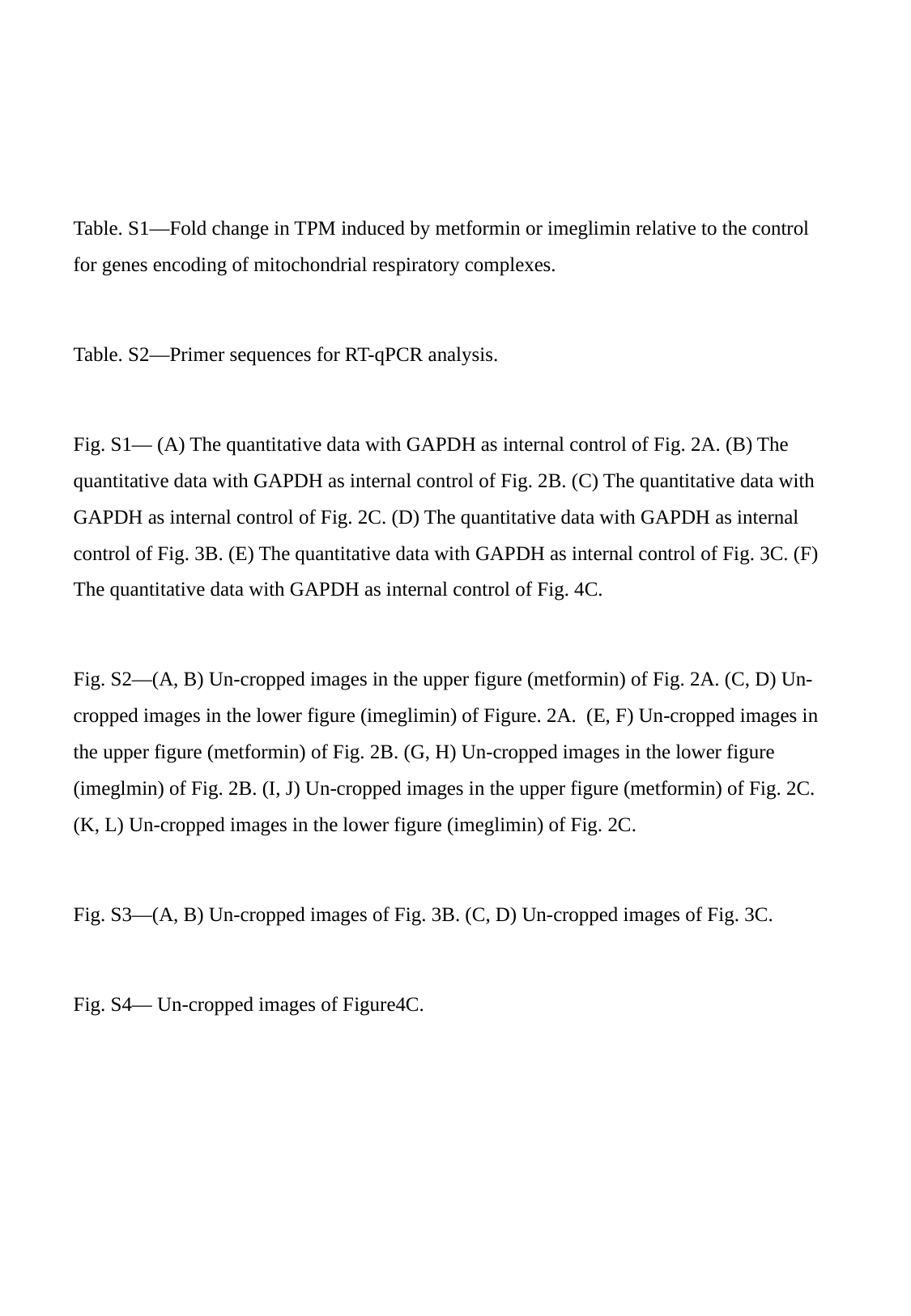

Table. S1—Fold change in TPM induced by metformin or imeglimin relative to the control for genes encoding of mitochondrial respiratory complexes.
Table. S2—Primer sequences for RT-qPCR analysis.
Fig. S1— (A) The quantitative data with GAPDH as internal control of Fig. 2A. (B) The quantitative data with GAPDH as internal control of Fig. 2B. (C) The quantitative data with GAPDH as internal control of Fig. 2C. (D) The quantitative data with GAPDH as internal control of Fig. 3B. (E) The quantitative data with GAPDH as internal control of Fig. 3C. (F) The quantitative data with GAPDH as internal control of Fig. 4C.
Fig. S2—(A, B) Un-cropped images in the upper figure (metformin) of Fig. 2A. (C, D) Un-cropped images in the lower figure (imeglimin) of Figure. 2A. (E, F) Un-cropped images in the upper figure (metformin) of Fig. 2B. (G, H) Un-cropped images in the lower figure (imeglmin) of Fig. 2B. (I, J) Un-cropped images in the upper figure (metformin) of Fig. 2C. (K, L) Un-cropped images in the lower figure (imeglimin) of Fig. 2C.
Fig. S3—(A, B) Un-cropped images of Fig. 3B. (C, D) Un-cropped images of Fig. 3C.
Fig. S4— Un-cropped images of Figure4C.

## Slide 3
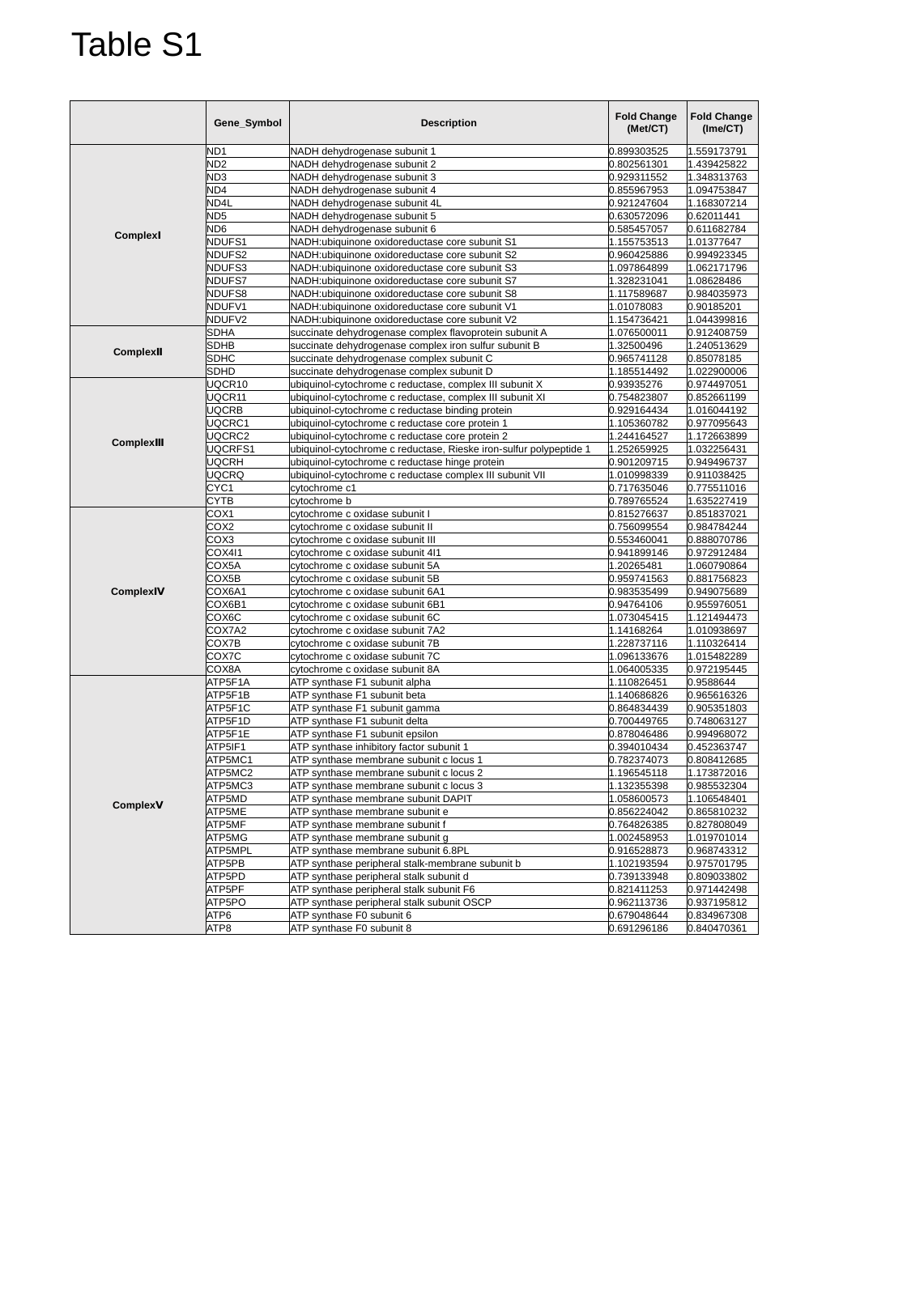

Table S1
| | Gene\_Symbol | Description | Fold Change (Met/CT) | Fold Change (Ime/CT) |
| --- | --- | --- | --- | --- |
| ComplexⅠ | ND1 | NADH dehydrogenase subunit 1 | 0.899303525 | 1.559173791 |
| | ND2 | NADH dehydrogenase subunit 2 | 0.802561301 | 1.439425822 |
| | ND3 | NADH dehydrogenase subunit 3 | 0.929311552 | 1.348313763 |
| | ND4 | NADH dehydrogenase subunit 4 | 0.855967953 | 1.094753847 |
| | ND4L | NADH dehydrogenase subunit 4L | 0.921247604 | 1.168307214 |
| | ND5 | NADH dehydrogenase subunit 5 | 0.630572096 | 0.62011441 |
| | ND6 | NADH dehydrogenase subunit 6 | 0.585457057 | 0.611682784 |
| | NDUFS1 | NADH:ubiquinone oxidoreductase core subunit S1 | 1.155753513 | 1.01377647 |
| | NDUFS2 | NADH:ubiquinone oxidoreductase core subunit S2 | 0.960425886 | 0.994923345 |
| | NDUFS3 | NADH:ubiquinone oxidoreductase core subunit S3 | 1.097864899 | 1.062171796 |
| | NDUFS7 | NADH:ubiquinone oxidoreductase core subunit S7 | 1.328231041 | 1.08628486 |
| | NDUFS8 | NADH:ubiquinone oxidoreductase core subunit S8 | 1.117589687 | 0.984035973 |
| | NDUFV1 | NADH:ubiquinone oxidoreductase core subunit V1 | 1.01078083 | 0.90185201 |
| | NDUFV2 | NADH:ubiquinone oxidoreductase core subunit V2 | 1.154736421 | 1.044399816 |
| ComplexⅡ | SDHA | succinate dehydrogenase complex flavoprotein subunit A | 1.076500011 | 0.912408759 |
| | SDHB | succinate dehydrogenase complex iron sulfur subunit B | 1.32500496 | 1.240513629 |
| | SDHC | succinate dehydrogenase complex subunit C | 0.965741128 | 0.85078185 |
| | SDHD | succinate dehydrogenase complex subunit D | 1.185514492 | 1.022900006 |
| ComplexⅢ | UQCR10 | ubiquinol-cytochrome c reductase, complex III subunit X | 0.93935276 | 0.974497051 |
| | UQCR11 | ubiquinol-cytochrome c reductase, complex III subunit XI | 0.754823807 | 0.852661199 |
| | UQCRB | ubiquinol-cytochrome c reductase binding protein | 0.929164434 | 1.016044192 |
| | UQCRC1 | ubiquinol-cytochrome c reductase core protein 1 | 1.105360782 | 0.977095643 |
| | UQCRC2 | ubiquinol-cytochrome c reductase core protein 2 | 1.244164527 | 1.172663899 |
| | UQCRFS1 | ubiquinol-cytochrome c reductase, Rieske iron-sulfur polypeptide 1 | 1.252659925 | 1.032256431 |
| | UQCRH | ubiquinol-cytochrome c reductase hinge protein | 0.901209715 | 0.949496737 |
| | UQCRQ | ubiquinol-cytochrome c reductase complex III subunit VII | 1.010998339 | 0.911038425 |
| | CYC1 | cytochrome c1 | 0.717635046 | 0.775511016 |
| | CYTB | cytochrome b | 0.789765524 | 1.635227419 |
| ComplexⅣ | COX1 | cytochrome c oxidase subunit I | 0.815276637 | 0.851837021 |
| | COX2 | cytochrome c oxidase subunit II | 0.756099554 | 0.984784244 |
| | COX3 | cytochrome c oxidase subunit III | 0.553460041 | 0.888070786 |
| | COX4I1 | cytochrome c oxidase subunit 4I1 | 0.941899146 | 0.972912484 |
| | COX5A | cytochrome c oxidase subunit 5A | 1.20265481 | 1.060790864 |
| | COX5B | cytochrome c oxidase subunit 5B | 0.959741563 | 0.881756823 |
| | COX6A1 | cytochrome c oxidase subunit 6A1 | 0.983535499 | 0.949075689 |
| | COX6B1 | cytochrome c oxidase subunit 6B1 | 0.94764106 | 0.955976051 |
| | COX6C | cytochrome c oxidase subunit 6C | 1.073045415 | 1.121494473 |
| | COX7A2 | cytochrome c oxidase subunit 7A2 | 1.14168264 | 1.010938697 |
| | COX7B | cytochrome c oxidase subunit 7B | 1.228737116 | 1.110326414 |
| | COX7C | cytochrome c oxidase subunit 7C | 1.096133676 | 1.015482289 |
| | COX8A | cytochrome c oxidase subunit 8A | 1.064005335 | 0.972195445 |
| ComplexⅤ | ATP5F1A | ATP synthase F1 subunit alpha | 1.110826451 | 0.9588644 |
| | ATP5F1B | ATP synthase F1 subunit beta | 1.140686826 | 0.965616326 |
| | ATP5F1C | ATP synthase F1 subunit gamma | 0.864834439 | 0.905351803 |
| | ATP5F1D | ATP synthase F1 subunit delta | 0.700449765 | 0.748063127 |
| | ATP5F1E | ATP synthase F1 subunit epsilon | 0.878046486 | 0.994968072 |
| | ATP5IF1 | ATP synthase inhibitory factor subunit 1 | 0.394010434 | 0.452363747 |
| | ATP5MC1 | ATP synthase membrane subunit c locus 1 | 0.782374073 | 0.808412685 |
| | ATP5MC2 | ATP synthase membrane subunit c locus 2 | 1.196545118 | 1.173872016 |
| | ATP5MC3 | ATP synthase membrane subunit c locus 3 | 1.132355398 | 0.985532304 |
| | ATP5MD | ATP synthase membrane subunit DAPIT | 1.058600573 | 1.106548401 |
| | ATP5ME | ATP synthase membrane subunit e | 0.856224042 | 0.865810232 |
| | ATP5MF | ATP synthase membrane subunit f | 0.764826385 | 0.827808049 |
| | ATP5MG | ATP synthase membrane subunit g | 1.002458953 | 1.019701014 |
| | ATP5MPL | ATP synthase membrane subunit 6.8PL | 0.916528873 | 0.968743312 |
| | ATP5PB | ATP synthase peripheral stalk-membrane subunit b | 1.102193594 | 0.975701795 |
| | ATP5PD | ATP synthase peripheral stalk subunit d | 0.739133948 | 0.809033802 |
| | ATP5PF | ATP synthase peripheral stalk subunit F6 | 0.821411253 | 0.971442498 |
| | ATP5PO | ATP synthase peripheral stalk subunit OSCP | 0.962113736 | 0.937195812 |
| | ATP6 | ATP synthase F0 subunit 6 | 0.679048644 | 0.834967308 |
| | ATP8 | ATP synthase F0 subunit 8 | 0.691296186 | 0.840470361 |
### Chart
| Category | Control | Metformin 250mg/kg | Imeglimin 250mg/kg |
|---|---|---|---|
| mHSPA1A | 1.0 | 0.6909035043674323 | 0.47089120549788 |
| mHSPA1B | 1.0 | 0.7042827905102651 | 0.4787460813879924 |
| mHSPH1 | 1.0 | 0.8562788563369644 | 0.6019298944463425 |
| mIGFBP3 | 1.0 | 0.8987910064160927 | 0.8711175800432892 |

## Slide 4
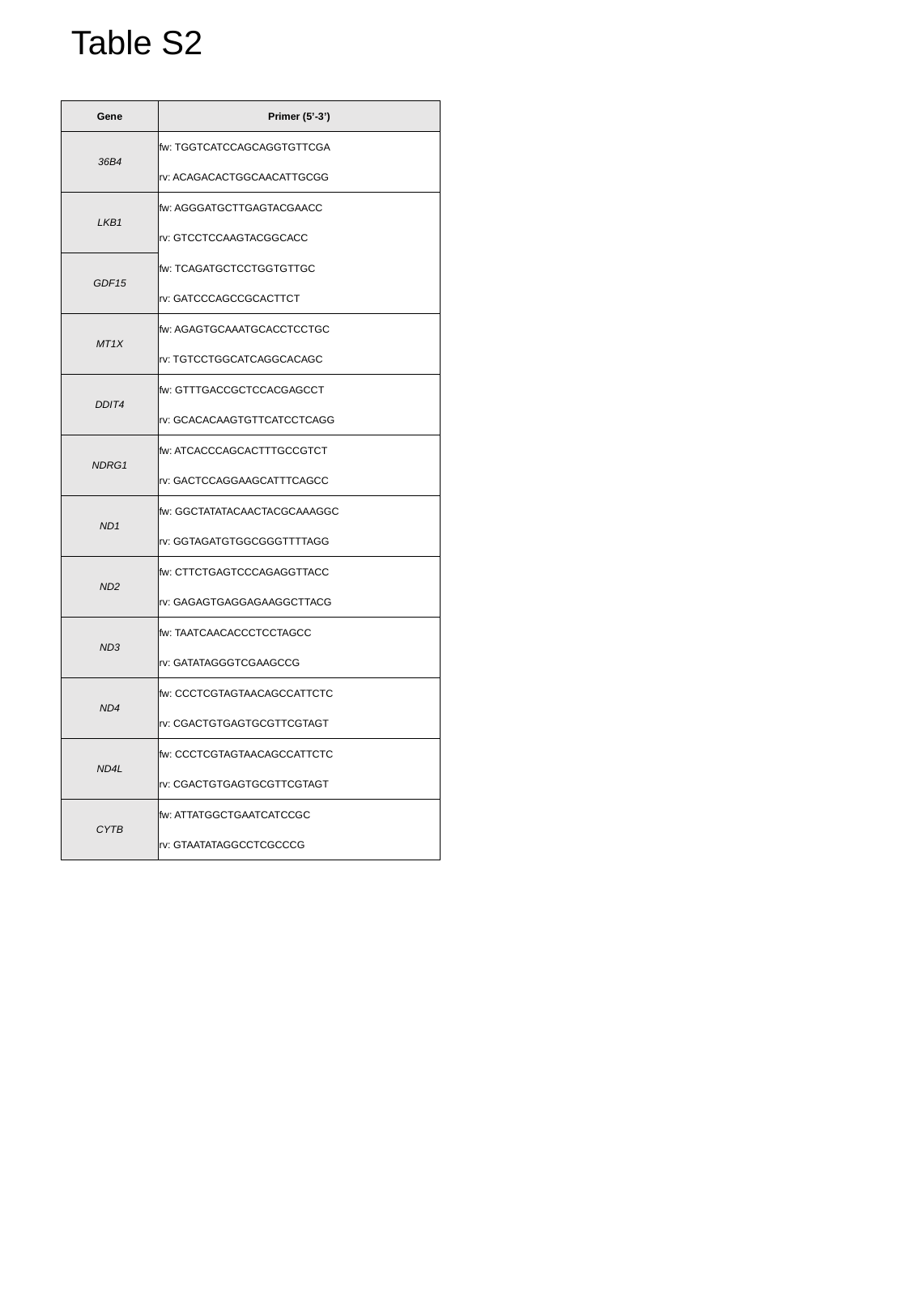

Table S2
| Gene | Primer (5’-3’) |
| --- | --- |
| 36B4 | fw: TGGTCATCCAGCAGGTGTTCGA |
| | rv: ACAGACACTGGCAACATTGCGG |
| LKB1 | fw: AGGGATGCTTGAGTACGAACC |
| | rv: GTCCTCCAAGTACGGCACC |
| GDF15 | fw: TCAGATGCTCCTGGTGTTGC |
| | rv: GATCCCAGCCGCACTTCT |
| MT1X | fw: AGAGTGCAAATGCACCTCCTGC |
| | rv: TGTCCTGGCATCAGGCACAGC |
| DDIT4 | fw: GTTTGACCGCTCCACGAGCCT |
| | rv: GCACACAAGTGTTCATCCTCAGG |
| NDRG1 | fw: ATCACCCAGCACTTTGCCGTCT |
| | rv: GACTCCAGGAAGCATTTCAGCC |
| ND1 | fw: GGCTATATACAACTACGCAAAGGC |
| | rv: GGTAGATGTGGCGGGTTTTAGG |
| ND2 | fw: CTTCTGAGTCCCAGAGGTTACC |
| | rv: GAGAGTGAGGAGAAGGCTTACG |
| ND3 | fw: TAATCAACACCCTCCTAGCC |
| | rv: GATATAGGGTCGAAGCCG |
| ND4 | fw: CCCTCGTAGTAACAGCCATTCTC |
| | rv: CGACTGTGAGTGCGTTCGTAGT |
| ND4L | fw: CCCTCGTAGTAACAGCCATTCTC |
| | rv: CGACTGTGAGTGCGTTCGTAGT |
| CYTB | fw: ATTATGGCTGAATCATCCGC |
| | rv: GTAATATAGGCCTCGCCCG |
### Chart
| Category | Control | Metformin 250mg/kg | Imeglimin 250mg/kg |
|---|---|---|---|
| mHSPA1A | 1.0 | 0.6909035043674323 | 0.47089120549788 |
| mHSPA1B | 1.0 | 0.7042827905102651 | 0.4787460813879924 |
| mHSPH1 | 1.0 | 0.8562788563369644 | 0.6019298944463425 |
| mIGFBP3 | 1.0 | 0.8987910064160927 | 0.8711175800432892 |

## Slide 5
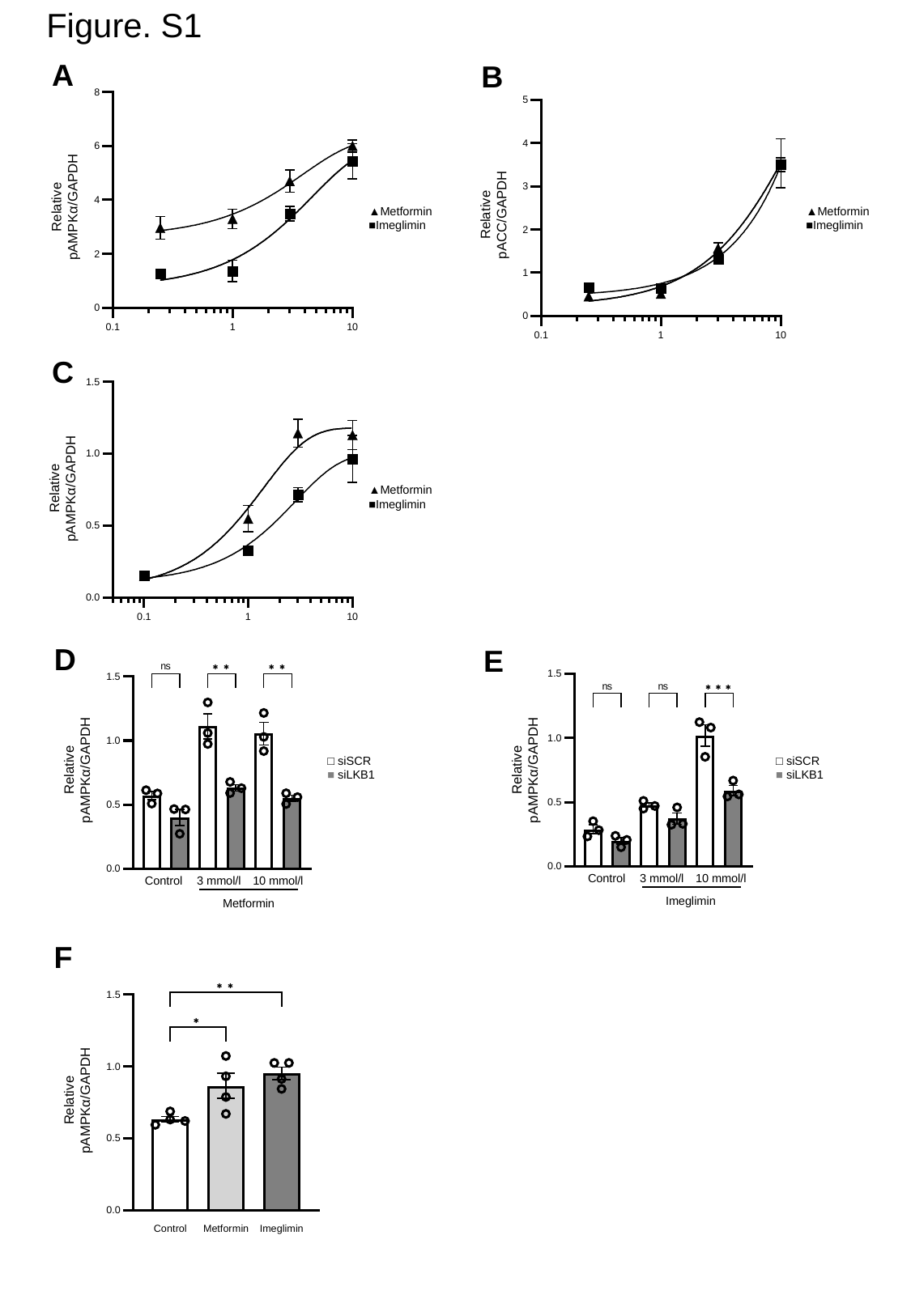

Figure. S1
A
B
Relative
pAMPKα/GAPDH
Relative
pACC/GAPDH
▲Metformin
■Imeglimin
▲Metformin
■Imeglimin
C
Relative
pAMPKα/GAPDH
▲Metformin
■Imeglimin
D
E
### Chart
| Category | Control | Metformin 250mg/kg | Imeglimin 250mg/kg |
|---|---|---|---|
| mHSPA1A | 1.0 | 0.6909035043674323 | 0.47089120549788 |
| mHSPA1B | 1.0 | 0.7042827905102651 | 0.4787460813879924 |
| mHSPH1 | 1.0 | 0.8562788563369644 | 0.6019298944463425 |
| mIGFBP3 | 1.0 | 0.8987910064160927 | 0.8711175800432892 |□ siSCR
■ siLKB1
□ siSCR
■ siLKB1
Relative
pAMPKα/GAPDH
Relative
pAMPKα/GAPDH
3 mmol/l
10 mmol/l
Control
3 mmol/l
10 mmol/l
Control
Imeglimin
Metformin
F
Relative
pAMPKα/GAPDH

## Slide 6
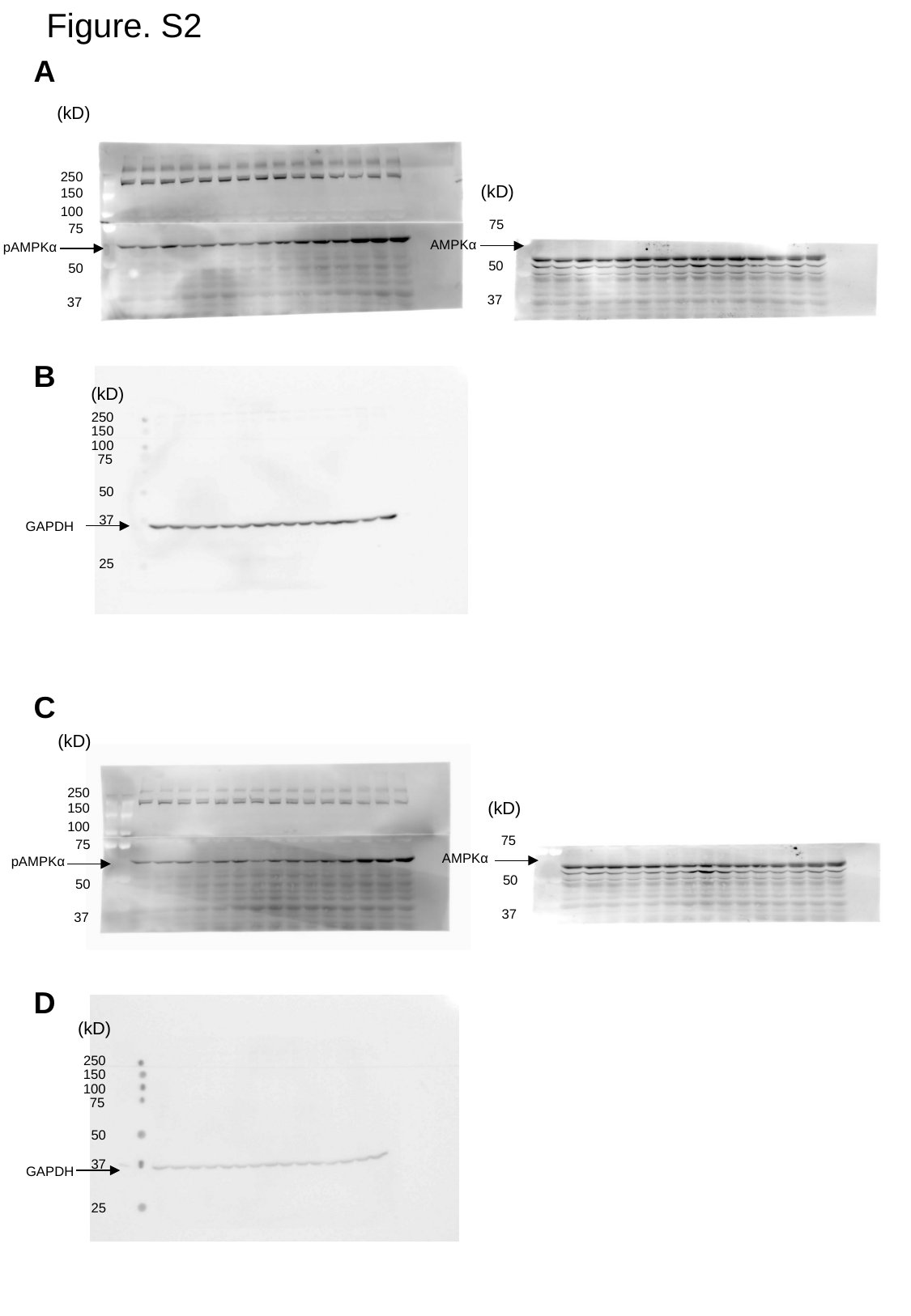

Figure. S2
A
(kD)
250
(kD)
150
100
75
75
AMPKα
pAMPKα
50
50
37
37
B
(kD)
250
150
100
75
50
37
GAPDH
25
C
(kD)
250
(kD)
150
100
75
75
AMPKα
pAMPKα
50
50
37
37
D
(kD)
250
150
100
75
50
37
GAPDH
25

## Slide 7
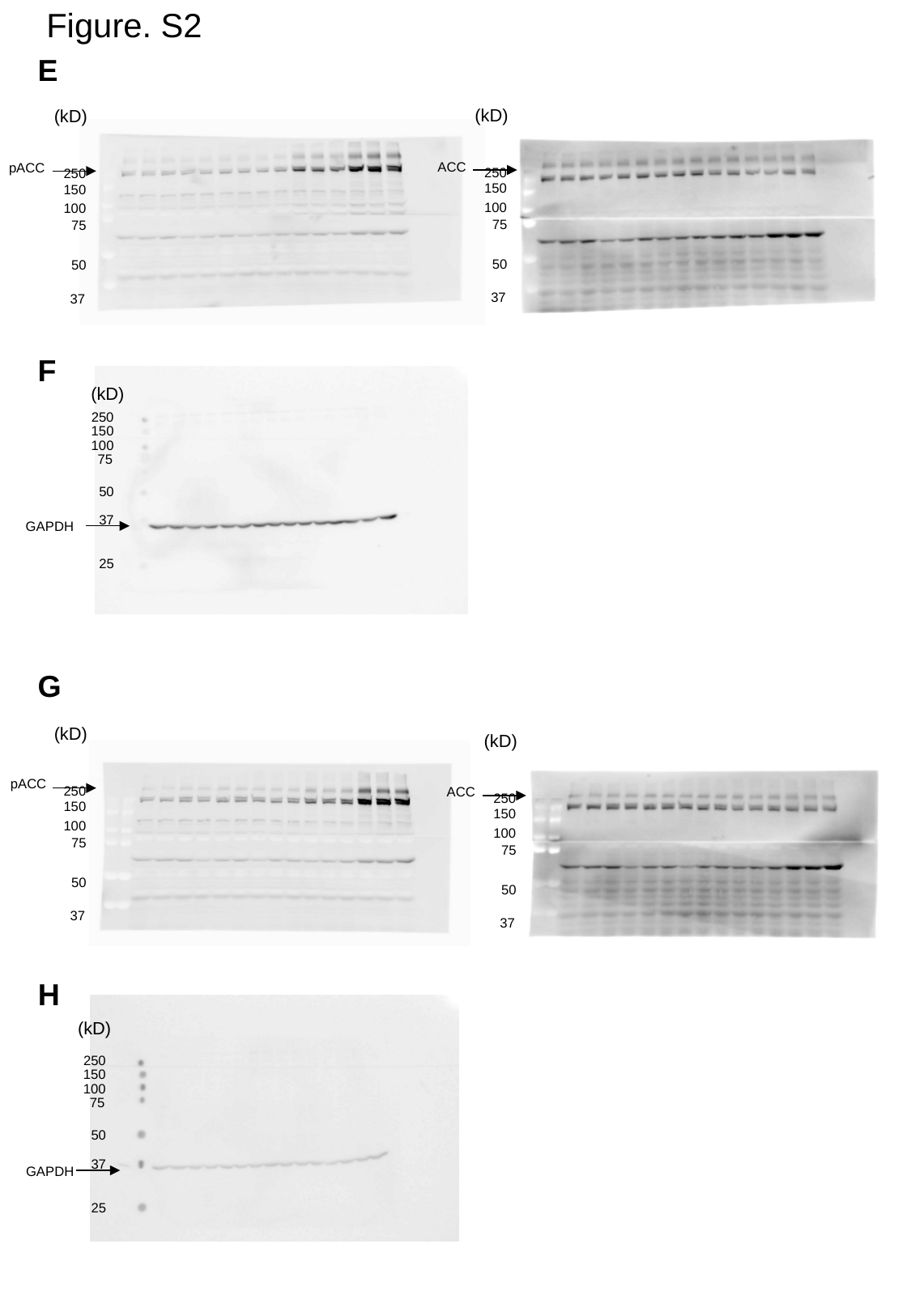

Figure. S2
E
(kD)
(kD)
ACC
pACC
250
250
150
150
100
100
75
75
50
50
37
37
F
(kD)
250
150
100
75
50
37
GAPDH
25
G
(kD)
(kD)
pACC
250
ACC
250
150
150
100
100
75
75
50
50
37
37
H
(kD)
250
150
100
75
50
37
GAPDH
25

## Slide 8
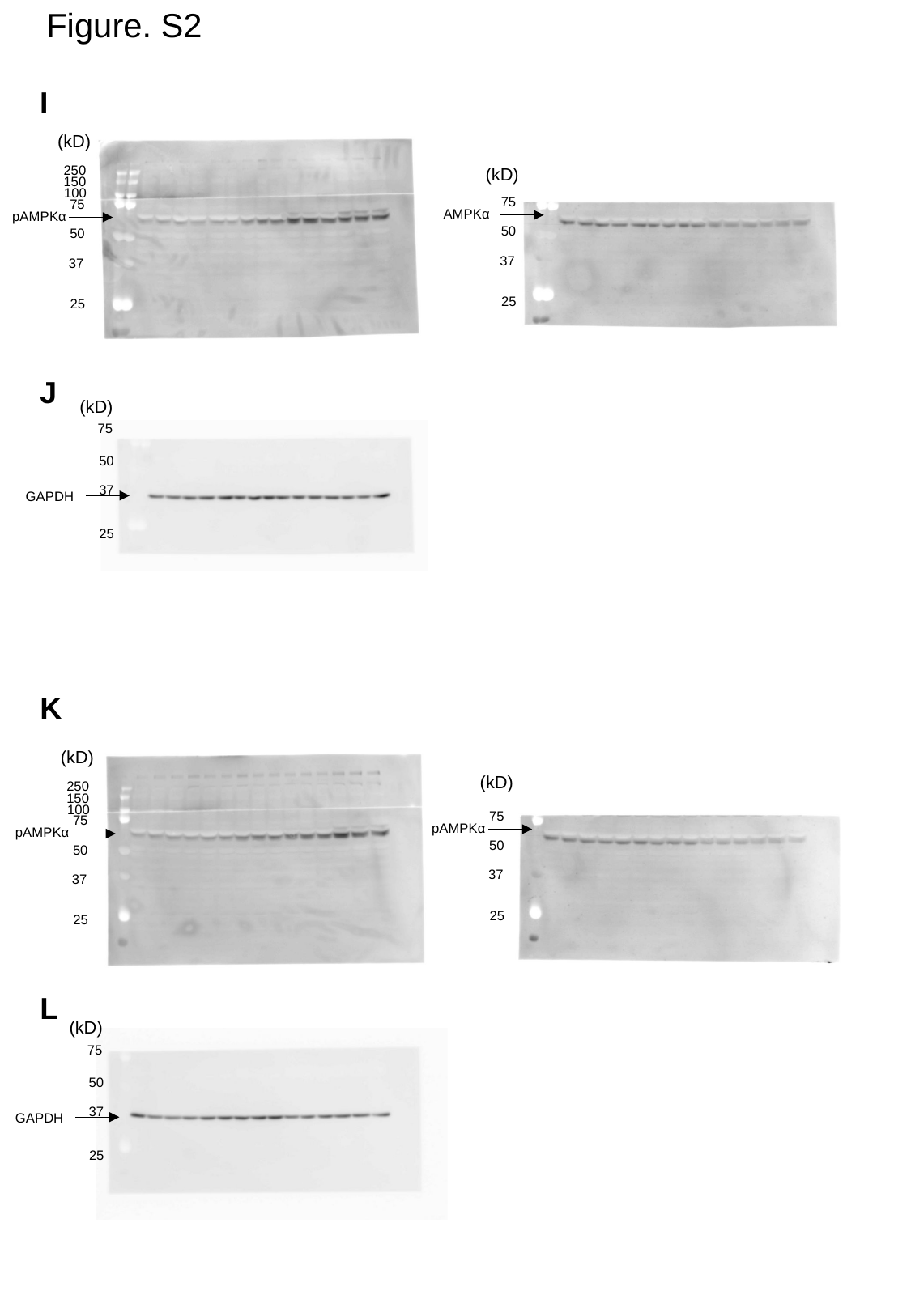

Figure. S2
I
(kD)
250
(kD)
150
100
75
75
AMPKα
pAMPKα
50
50
37
37
25
25
J
(kD)
75
50
37
GAPDH
25
K
(kD)
(kD)
250
150
100
75
75
pAMPKα
pAMPKα
50
50
37
37
25
25
L
(kD)
75
50
37
GAPDH
25

## Slide 9
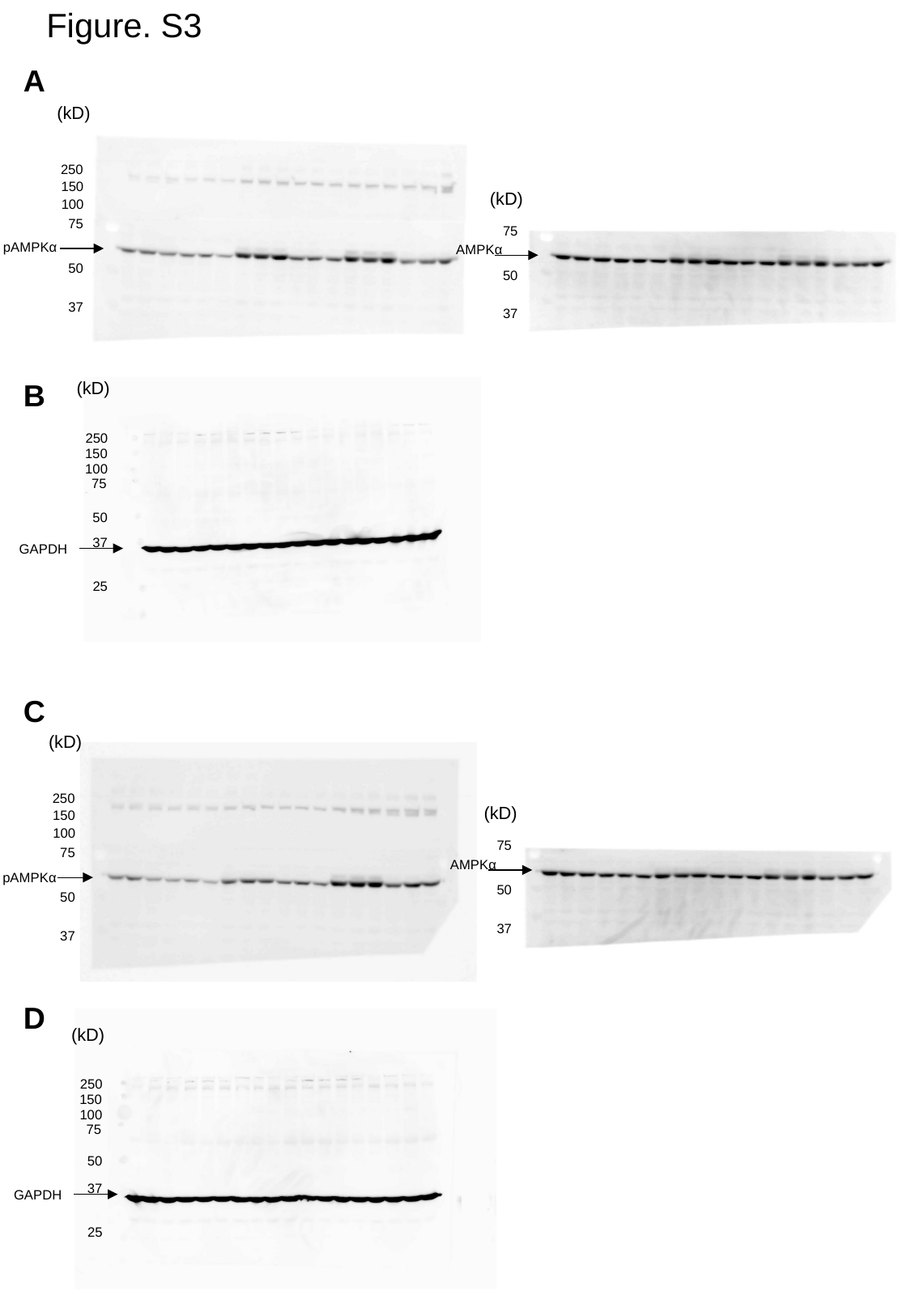

Figure. S3
A
(kD)
250
150
(kD)
100
75
75
pAMPKα
AMPKα
50
50
37
37
B
(kD)
250
150
100
75
50
37
GAPDH
25
C
(kD)
250
(kD)
150
100
75
75
AMPKα
pAMPKα
50
50
37
37
D
(kD)
250
150
100
75
50
37
GAPDH
25

## Slide 10
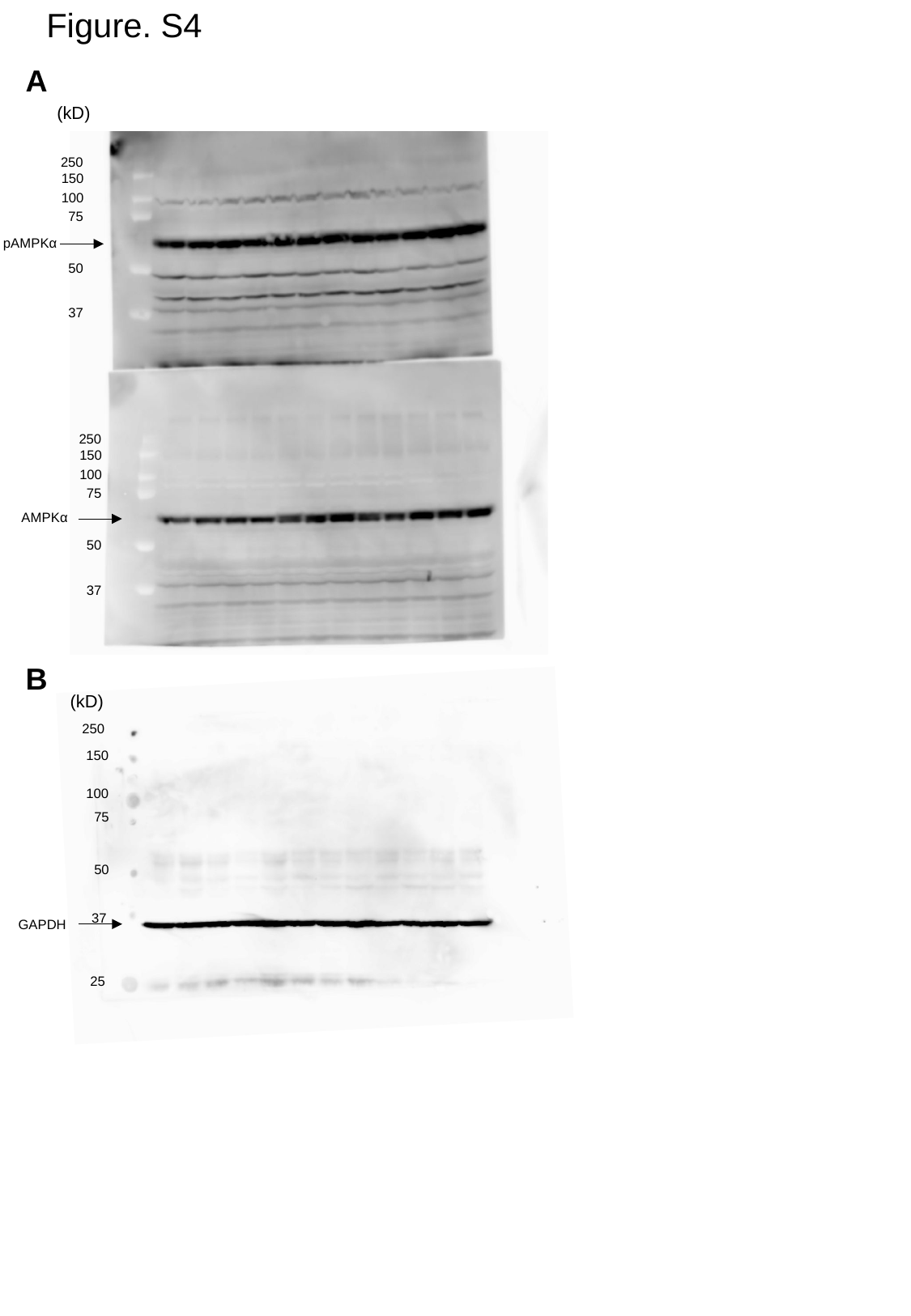

Figure. S4
A
(kD)
250
150
100
75
pAMPKα
50
37
250
150
100
75
AMPKα
50
37
B
(kD)
250
150
100
75
50
37
GAPDH
25
